# Induced Dryness Stress on Human Vaginal Epithelium: The Efficacy of a New Vaginal Gel

**DOI:** 10.3390/gels7040157

**Published:** 2021-09-28

**Authors:** Guglielmo Stabile, Giuseppe Ricci, Maria Sole Scalia, Francesco De Seta

**Affiliations:** 1 Institute for Maternal and Child Health—IRCCS “Burlo Garofolo”, 34137 Trieste, Italy; giuseppe.ricci@burlo.trieste.it (G.R.); fradeseta@gmail.com (F.D.S.); 2 Department of Medicine, Surgery and Health Sciences, University of Trieste, 34127 Trieste, Italy; mariasole.scalia@hotmail.it

**Keywords:** vulvovaginal atrophy, genitourinary syndrome, menopause, vaginal gel, dryness

## Abstract

An experimental model of dryness on vaginal mucosa is proposed to assess the efficacy of a new vaginal gel (Respecta^®^ Hydragel Ref 17031). The dryness model was induced on reconstituted human vaginal epithelium (HVE) by incubating the tissues in modified environmental conditions (R.H. < 50% and T = 40 °C) for 48 h. The products were applied on the ‘Dry’ HVE models for 24 h (series 48 h + 24 h) in standard culture conditions (37 °C 5% CO_2_). Their efficacy in counteracting vaginal dryness was assessed and compared to tissues treated with saline solution and cultured in standard culture conditions (negative control) and to untreated tissues incubated in dryness conditions for 48 h and then recovered after 24 h in standard culture conditions (positive control). The products’ efficacy was quantified by measuring the following parameters: (1) water flux and direct moisturization by AQP3 immunohistochemical staining, and (2) maintenance of moisturization and elasticity of the mucosa by hyaluronic acid (CD44) immunofluorescence staining. Respecta^®^ Hydragel demonstrated efficacy in regulating the water flux by inducing AQP3 expression thus determining a positive water balance within the vaginal epithelium. It induced a remodelling of the epithelium morphology with restored trophism compared to the dry HVE control. Furthermore, it demonstrated a significant increase of the expression of CD44, related to hyaluronic acid (HA) distribution in the extracellular matrix. HA has the ability to act on the cellular matrix composition and its renewal compared to the dry HVE control. Through these mechanisms it induces a deep hydration and elasticity of the vaginal mucosa.

## 1. Introduction

Menopause generally arises in the fourth or fifth decade of life resulting in lower levels of circulating estrogen. Such deficit is accountable for symptoms such as hot flushes, night sweats, decreased cognitive functions, mood changes and genitourinary tract alterations associated with vulvovaginal atrophy (VVA). The modifications of the vulvovaginal district are common and can be found in at least 50% of menopausal women [1,2,3,4,5]. In VVA there can be found many signs and the most common include dryness, redness, itching, pain at intercourse, and occasionally discharge or bleeding [6,7] Until now and for the past decades, the therapy mostly employed for the management of VVA and vaginal dryness in post menopausal women has been either systemic or with local low-dose estrogen therapy [8,9,10,11]

Because of the chronic nature of VVA and genitourinary syndrome of menopause (GSM), treatments are recommended to be prescribed at the onset of the symptoms and signs of atrophic changes of the vagina in order to avoid the development of severe pictures of the condition, and should be continued over time so as to maintain their benefits [12]. The therapy needs to be targeted and personalized, taking into account the preferences of women. In fact, the level of comfort with a given therapy is strongly influenced by both individual and socioenvironmental factors. Nonhormonal strategies may be used in women of any age in which either hormonal treatments are contraindicated or as a co-treatment for those already prescribed with systemic/vaginal hormone therapy. The prescription of vaginal moisturizers and lubricants together with the continuation of sexual activity may be helpful in improving their living standards.

In literature, there can be found few clinical trials that have proved the efficacy of such products. Lubricants are substances that act quickly, mostly based on water, silicone or oil and are useful to reduce friction during sexual activity. On the other hand, moisturizers are longer acting and may provide a trophic effect [12]. 

In vitro reconstructed human epithelial models are a way to test the effectiveness of topical products. These models are closer to in vivo human tissues thank to their morphology (multistratified or epithelium), and to their biochemical and physiological properties and represent today an unique test system that permits the study of skin biology mechanism and the set-up of experimental models that can lead to the quantification of ingredients and products mechanism of action together with their related efficacy. Furthermore, the presence of an organized tissue with different cell layers allows to apply the products topically at the same doses than in vivo increasing the biological relevance and predictivity of 3D tissue based approaches as compared to cell culture based systems. 

In our study, we tested the following two products on the above-mentioned models:

1. RESPECTA^®^ HYDRAGEL (Ref. 17031): active compounds in a mucoadhesive matrix with excipients.
2. REFERENCE OR PLACEBO GEL: mucoadhesive matrix with excipients.

## 2. Results

An experimental model of dryness on vaginal mucosa was proposed to assess the efficacy of protective, hydrating and soothing products in reducing the symptoms associated with major loss of water content at mucosal level. The tissue used for the experiments is not an expiant for human subjects; it is a commercial reconstituted human vaginal epithelium (HVE) purchased from EPISKIN Laboratoires, 4 Rue Alexander Fleming 69366 Lyon (France). This model is a multistratified tissue (>six layers) and it reproduces vaginal epithelium morphology i.e., an epithelium of 60–90 μm formed after 5 days of air-lift culture of immortalized cell line (A431) [13]. 

The product involved in our study was Respecta^®^ Hydragel Ref. 17031, a medical device (class IIa) for local administration manufactured by Giellepi S.P.A. indicated for the prevention and treatment of VVA. It is based on active compounds of hyaluronic acid, lactic acid, VGF^®^ (germinated Oryza sativa grain) and polydextrose. This product was compared to the placebo gel composed of the same ingredients (EDTA, Carbopol, Xanthan gum, Farnesol) without the defined active compounds. 

**Figure 1:**
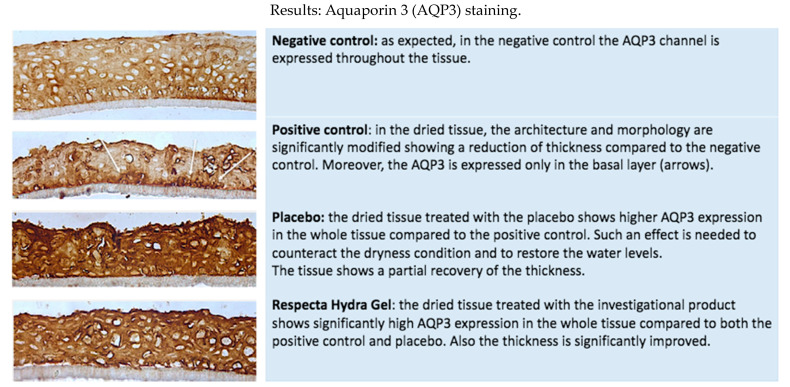
Histological analysis with immunohistochemical stain.

**Figure 2:**
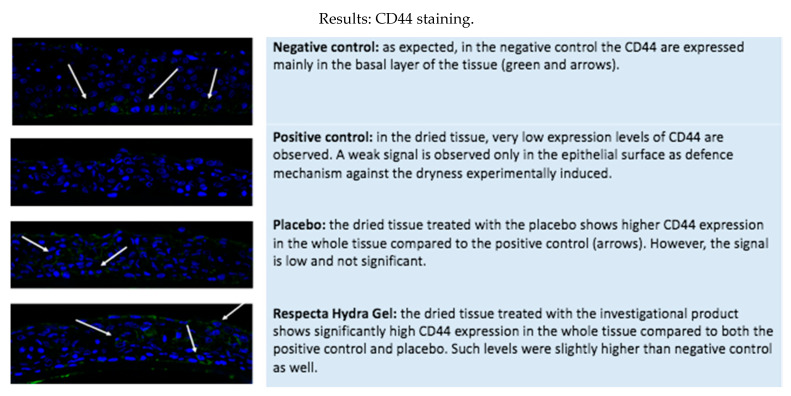
Immunofluorescence staining (CD-44) related to hyaluronic acid distribution.

In order to do so, we evaluated four tissue models: a negative control (HVE tissue treated with 10 μL of saline solution and incubated in standard culture conditions for 48 h) was compared to a positive control (tissues incubated in dryness culture conditions for 48 h, then recovered in standard conditions after 24 h corresponding to the time of exposures in the treated serie) and to two treated tissues, respectively with Respecta Hydragel and with placebo. (Figure 1 and Figure 2)

The theoretical advantages of the formulation object of the study should be the moisturizing and lubricating action related to the active compounds:

1. Hyaluronic acid should protect and hydrate the tissue;
2. Lactic acid reduced pH value (acidic pH is physiologic in vagina);
3. VGF^®^ (germinated Oryza sativa grain) has a soothing effect;
4. Polydextrose should support bacterial growth (lactobacilli are dominant in healthy situation).

Three vertical tissue sections are prepared on each histological slide; on one selected slide three microscopical acquisitions of three selected parts were performed. For each sample is reported the most representative acquisition of the selected vertical section.

Product efficacy was quantified by measuring:

(1) Water flux and direct moisturization by AQP3 immunohistochemical staining (Table 1, Figure 1 and Figure 2).
(2) Maintenance of moisturization and elasticity of the mucosa by HA (CD44) immunofluorescence staining (Table 1, Figure 1 and Figure 2).

Respecta^®^ Hydragel is effective in regulating the water flux by inducing AQP3 expression thus determining a positive water balance within the vaginal epithelium. It leads to a significant increase of the expression of CD44, related to hyaluronic acid (HA) distribution with the extracellular matrix inducing a deep hydration and elasticity of the vaginal mucosa.

## 3. Discussion

Genitourinary syndrome of menopause is very prevalent, underdiagnosed, and inadequately treated. Whereas vasomotor symptoms often diminish overtime, GSM is unlikely to resolve spontaneously, and often progresses if not treated. Due to increasing longevity, women may now suffer from GSM for more than one third of their life. Vaginal dryness, in particular, affects more or less 50% of postmenopausal women [14]. The impact of vaginal dryness on the quality of life is often underestimated. From the perspective of the couple, vaginal dryness can cause relationship issues. It can accentuate erectile dysfunction in the male partner, and even cause single women to avoid entering into a relationship with a potential new partner [15]. The choice of a specific therapy depends on symptoms severity, on treatment effectiveness and safety, and on patient preferences [16].

Estrogen therapy, either vaginal, in low doses, or systemic, remains the therapeutic standard for symptomatic women who suffer from moderate to severe GSM, and for those who do not sufficiently improve with the use of lubricants or moisturizers.

However, as suggested by the widely accepted international standards for the treatment of mild and moderate manifestations of VVA, first-line treatments are nonhormonal vaginal lubricants that should be used before intercourse and vaginal moisturizers with a long-term effect with frequent use (several times a week). In these cases, the endurance of regular sexual activity is to be taken into account [17].

The main difference between vaginal lubricants and moisturizers is the timing of application. Vaginal lubricants are particularly indicated for women whose main concern is vaginal dryness during intercourse. Lubricants can provide short-term relief from vaginal dryness and discomfort during sexual intercourse. These products are based on water, silicone, or oil and are locally applied to the vulva, vagina, or penis right before sexual activity. Water based lubricants are convenient when compared to lubricants based on silicone, because they have less side effects [15].

Vaginal moisturizers are used daily or every 2 to 3 days (depending on the symptom severity) in order to maintain vaginal hydration. They have a long-lasting effect in relieving the symptoms of VVA, intensifying vaginal mucosa moisture, and reducing the local pH. They consist of insoluble hydrophilic cross-linked polymers with a characteristic bio-adhesiveness that is able to adhere to the epithelium of the vaginal wall by retaining water. They may contain a decent amount of excipients that influence the pH and the osmolality of the formulation [18]. The most popular are based on hyaluronic acid (HA), a glycosaminoglycan produced by fibroblasts, which is the main component of the extracellular matrix. The mechanism by which hyaluronic acid works is through its capacity to bind water, which eventually leads to cellular movement and thus cell migration [19]. Accordingly, in case of tissue damage, HA stimulates the migration and proliferation of fibroblasts which are responsible for the deposit of collagen fibers, and it promotes neo-angiogenesis and tissue regeneration.

According to Chen et al. [20], the use of hyaluronic acid vaginal gel every 3 days can be compared to the same improvement in symptoms of vaginal dryness given by local estrogen therapy.

Women who have a legit contraindication to estrogen or those who choose not to use estrogen gain clear benefits from the use of a good-quality lubricant/moisturizer. However, in our view, a good-quality lubricant produces benefits to most women and couples regardless of whether it is appropriate for the woman to use estrogen or not.

Our experiments showed that Respecta^®^ Hydragel has a vaginal moisturizer and lubricant function and is specially designed for menopausal women with vulvar vaginal atrophy and dryness. The richness in water of the extracellular matrix corresponds to turgidity of the mucosa that cooperates with the supporting function of well-structured epithelium. The effect on the epithelium trophism and the activity on the moisturization and elasticity of the matrix is evaluated by targeting AQP3 and hyaluronic acid (expression of CD44), substances able to regulate and preserve water molecules. Respecta^®^ Hydragel is a safe product whose reliability is proven by the lack of morphological alterations after its use on the HVE samples, as reflected by histological evidence.

Further clinical studies are anyhow needed to confirm the positive in vitro results.

## 4. Conclusions

Vaginal dryness is a common symptom in women with vulvovaginal atrophy or genitourinary syndrome of menopause and has a substantial negative impact on their sexual and overall quality of life [15]. Personal lubricants and moisturizers are effective treatment options in the management of vaginal dryness with a variety of causes and can be used as a first-line treatment [21]. However, differences exist between commercially available products.

Respecta^®^ Hydragel has demonstrated efficacy in regulating the water flux by inducing AQP3 expression thus determining a positive water balance within the vaginal epithelium. It induced a remodelling of the epithelium morphology with restored tropism compared to the dry HVE control. Furthermore, it demonstrated a significant increase of the expression of CD44, related to hyaluronic acid (HA) distribution in the extracellular matrix. It has the ability to act on the cellular matrix composition and its renewal compared to the dry HVE control. Through these mechanisms it induces a deep hydration and elasticity of the vaginal mucosa.

## 5. Materials and Methods

The dryness model was induced on reconstituted human vaginal epithelium (HVE) by incubating the tissues in modified environmental conditions (R.H. < 50% and T = 40 °C) for 48 h. The resulting model mimicks the discomfort associated with transient inflammation and a dryness condition associated with reduced barrier function: these symptoms are often described as “irritation”.

We tested two products:

1. RESPECTA^®^ HYDRAGEL (Ref. 17031, Giellepi, Milano, Italy): active compounds (hyaluronic acid, lactic acid, VGF^®^-germinated Oryza sativa grain, polidextrose) in a mucoadhesive matrix (carbopol, xanthan gum) with excipients;
2. REFERENCE OR PLACEBO GEL (Giellepi, Milano, Italy) mucoadhesive matrix (carbopol, xanthan gum) with excipients.

The products were applied on the ‘DRY’ HVE models for 24 h (series 48 h + 24 h) in standard culture conditions (37 °C 5% CO_2_) and their efficacy in counteracting vaginal dryness was assessed and compared to tissues treated with saline solution and cultured in standard culture conditions (negative control) and untreated tissues incubated in dryness conditions for 48 h and then recovered after 24 h in standard culture conditions (positive control).

Product efficacy was quantified by measuring:

(1) Water flux and direct moisturization by AQP3 immunohistochemical staining (Table 1);
(2) Maintenance of moisturization and elasticity of the mucosa by hyaluronic acid using CD44 immunofluorescence staining (Table 1).

### 5.1. Dryness Model Establishment

The day of arrival, HVE tissues were incubated for 2–3 h in standard culture conditions (37 °C 5% CO_2_).

The negative control was treated with 10 μL of saline solution and incubated in standard culture conditions for 48 h (three tissues). The positive control and treated tissues were placed in a CO_2_ incubator in modified environmental conditions mimicking a dryness stress (R.H. < 65% and T = 40 °C) for 48 h.

### 5.2. Sample Treatment

After 48 h of dryness, 20 μL of test items were applied topically for 24 h (series 48 h + 24 h) on the HVE cultured in standard conditions (three tissues).

For the negative control, after 48 h in standard culture conditions, the tissues were recovered after 24 h in the same culture conditions (three tissues).

For the positive control, after 48 h in dryness conditions, the tissues were recovered after 24 h in standard culture conditions (three tissues).

The following parameters were assessed after 24 h recovery in order to quantify a real benefit in terms of homeostasis restoring mediated by different mechanisms:

(1) Water flux and direct moisturization by AQP3 immunohistochemical staining;
(2) Maintenance of moisturization and elasticity of the mucosa by HA (CD44) immunofluorescence staining.

### 5.3. Test System

EPISKIN reconstituted human vaginal epithelium (HVE) of 0.5 cm^2^ (manufactured by EPISKIN Laboratoires, Lyon, France) was used.

The model reproduces vaginal epithelium morphology and it was fully characterized: an epithelium of 60–90 μm was formed after 5 days of air-lift culture of immortalized cell line (A431) in a chemically defined medium. The tissue and media were manufactured in compliance with ISO 9001. The intended use of the biological model is for research purposes only (standardized in vitro testing of chemicals or formulations).

The HVE batch was tested for the absence of HIV, hepatitis B, hepatitis C and mycoplasma. The maintenance medium was tested for sterility. The inserts containing the HVE at day 5 were shipped at room temperature in a multiwell plate filled with an agarose nutrient solution in which they were embedded.

### 5.4. Media and Cultures Conditions

Immediately after arrival in the laboratory, data sheets enclosed to the batch, shipment integrity, colour and temperature of the agar medium used for transport were checked. The HVE was then removed from the agarose nutrient solution under a sterile air flow cabin. The inserts were rapidly transferred to a six-well plate previously filled with a maintenance medium (1 mL/well) at room temperature and incubated at 37 °C, 5% CO_2_, saturated humidity.

Negative control was the saline solution (0,9% NaCl) in which neutral action on epithelial tissues was demonstrated.

Positive control were tissues incubated in dryness culture conditions (CO_2_ incubator with R.H. < 50% and T = 40 °C) for 48 h and recovered in standard conditions (37 °C and 5% CO_2_) after 24 h corresponding to the time of exposures in the treated series.

### 5.5. Immuno-Histology: Aqp3 Detection

The histological evaluation is a complementary endpoint, useful to confirm the biochemical investigations and for a deeper understanding of the type of the interaction between the product and the living tissue.

Aquaporins (AQPs) are proteins located on the cell membrane of the most of human tissues and organs. They create channels on the cell membrane, which can control the flow of water through the cell membrane. To date, 13 family members (AQP0-AQP12) have been already identified but the AQP3 is the best characterized. In dried tissues (like skin or mucosa) AQP expression usually increases in order to counterbalance the dryness and restore the physiological tissue moisture. Preclinical evidence sustains such an action. AQP3 deficient mice displayed reduced water holding capacity in the epidermis which cannot be improved by increased environmental humidity or occlusion [22,23].

Paraffin slides were deparaffinized and rehydrated, then 8 min at 99 °C incubation in citrate buffer was performed as antigen retrieval, then quenching of peroxidase with 3% H_2_O_2_ in MetOH for 10 min at room temperature followed by incubation overnight with primary antibody AQP3 rabbit polyclonal (1:120 in 1% BSA in PBS, Abcam 153694). After 15 min of incubation with HRP, the slides were incubated with DAB (Power Stain 1.0 poly HRP DAB kit, Genemed, Torrance, CA, USA).

The histological samples have been analyzed under light microscopy (40×): the overall morphology and its modification compared to the negative controls were analyzed on at least three sections of the same tissue and reported in the laboratory notebook.

### 5.6. Immuno-Fluorescence: CD44 Detection

Immunofluorescence (IF) labelling is an histological technique to detect specific molecules or structures in cellular compartments of histological sections. The technique is based on the specificity of the antigen-binding antibody for the detection of the target molecule and of a detection system by fluorescence microscopy with indirect method.

Hyluronic acid (HA) is a high molecular weight glycosaminoglycan, which is a major component of the extracellular matrix and retains water effectively, absorbing >1000 times its weight (Yao et al. 2021). Due to its capability to retain high amount of water, HA is responsible to facilitate tissue hydration, and positively influence some cellular key processes such as proliferation and wound heling. HA interacts with CD44 expressed on the cell surface, thus creating a link between cells and extracellular matrix. The higher expression of CD44 is linked to higher HA molecules retained at cellular level with consequent positive effect on the epithelial/tissues moisture and hydration.

The immuno-fluorescence labeling was performed according to VitroScreen procedure P M 13. For immunostaining, the following primary and secondary antibodies were used:

Anti-CD44 Cell Signalling 1563C11.

Alexa Fluor 488 Goat antimouse Lifetechnologies A10680.

## Figures and Tables

**Table 1:** Mechanism of action and results of the product’s application after histomorphological analysis.

	AQP3 HIC	CD44 IF
RESPECTA^®^ GEL IDRATANTE VAGINALE Ref. 17031 (A)	Efficacy of the product in regulating the water flux by inducing AQP3 expression thus determining a positive water balance within the vaginal epithelium. Remodelling of the epithelium morphology with restored trophism compared to the dry HVE control.	Significant increase of the expression of CD44, related to hyaluronic acid (HA) distribution with the extracellular matrix and inducing a deep hydration and elasticity of the vaginal mucosa. Efficacy in regulating the extracellular matrix composition and renewal compared to the DRY HVE control.
REFERENCE (B)	Efficacy of the reference in regulating the water flux by modulating AQP3 expression thus confirming its principal activity.	Lower effect compared to the product Ref. 17031

## Data Availability

All raw data are stored in files/hard copies registered in VitroScreen server/archive and are available.
